# *RNF43* mutations predict response to anti-BRAF/EGFR combinatory therapies in *BRAF*^V600E^ metastatic colorectal cancer

**DOI:** 10.1038/s41591-022-01976-z

**Published:** 2022-09-12

**Authors:** Elena Elez, Javier Ros, Jose Fernández, Guillermo Villacampa, Ana Belén Moreno-Cárdenas, Carlota Arenillas, Kinga Bernatowicz, Raquel Comas, Shanshan Li, David Philip Kodack, Roberta Fasani, Ariadna Garcia, Javier Gonzalo-Ruiz, Alejandro Piris-Gimenez, Paolo Nuciforo, Grainne Kerr, Rossana Intini, Aldo Montagna, Marco Maria Germani, Giovanni Randon, Ana Vivancos, Ron Smits, Diana Graus, Raquel Perez-Lopez, Chiara Cremolini, Sara Lonardi, Filippo Pietrantonio, Rodrigo Dienstmann, Josep Tabernero, Rodrigo A. Toledo

**Affiliations:** 1 Medical Oncology Department, Vall d’Hebron University Hospital, Vall d’Hebron Barcelona Hospital Campus, Barcelona, Spain; 2 Vall d’Hebron Institute of Oncology (VHIO), Vall d’Hebron Barcelona Hospital Campus, Barcelona, Spain; 3 Oncologia Medica, Dipartimento di Medicina di Precisione, Università degli Studi della Campania Luigi Vanvitelli, Naples, Italy; 4 Centro de Investigación Biomédica en Red de Cáncer (CIBERONC), Institute of Health Carlos III (ISCIII), Madrid, Spain; 5 Department of Gastroenterology and Hepatology, Erasmus MC-University Medical Center, Rotterdam, the Netherlands; 6 Novartis Institutes for BioMedical Research, Cambridge, MA USA; 7 Oncology Department, Novartis Institutes for Biomedical Research, Novartis, Basel, Switzerland; 8 Department of Oncology, Veneto Institute of Oncology IRCCS, Padova, Italy; 9 Unit of Medical Oncology, Azienda Ospedaliero-Universitaria Pisana, Department of Trans-lational Research and New Technologies in Medicine, University of Pisa, Pisa, Italy; 10 Department of Medical Oncology, Fondazione IRCCS Istituto Nazionale dei Tumori, Milan, Italy; 11 Radiology Department, Vall d’Hebron University Hospital, Vall d’Hebron Barcelona Hospital Campus, Barcelona, Spain; 12 UVic-UCC, IOB-Quirón, Barcelona, Spain; 13 Present Address: Ridgeline Discovery, Basel, Switzerland

**Keywords:** Colorectal cancer, Tumour biomarkers, Predictive markers

## Abstract

Anti-BRAF/EGFR therapy was recently approved for the treatment of metastatic *BRAF*^V600E^ colorectal cancer (mCRC^BRAF-V600E^). However, a large fraction of patients do not respond, underscoring the need to identify molecular determinants of treatment response. Using whole-exome sequencing in a discovery cohort of patients with mCRC^BRAF-V600E^ treated with anti-BRAF/EGFR therapy, we found that inactivating mutations in *RNF43*, a negative regulator of WNT, predict improved response rates and survival outcomes in patients with microsatellite-stable (MSS) tumors. Analysis of an independent validation cohort confirmed the relevance of *RNF43* mutations to predicting clinical benefit (72.7% versus 30.8%; *P* = 0.03), as well as longer progression-free survival (hazard ratio (HR), 0.30; 95% confidence interval (CI), 0.12–0.75; *P* = 0.01) and overall survival (HR, 0.26; 95% CI, 0.10–0.71; *P* = 0.008), in patients with MSS-*RNF43*^mutated^ versus MSS-*RNF43*^wild-type^ tumors. Microsatellite-instable tumors invariably carried a wild-type-like *RNF43* genotype encoding p.G659fs and presented an intermediate response profile. We found no association of *RNF43* mutations with patient outcomes in a control cohort of patients with MSS-mCRC^BRAF-V600E^ tumors not exposed to anti-BRAF targeted therapies. Overall, our findings suggest a cross-talk between the MAPK and WNT pathways that may modulate the antitumor activity of anti-BRAF/EGFR therapy and uncover predictive biomarkers to optimize the clinical management of these patients.

## Abstract (web summary)

The presence of inactivating mutations in *RNF43*, a negative regulator of WNT, in tumor cells predicts improved response rates and survival outcomes in patients with metastatic *BRAF*^V600E^ colorectal cancer treated with anti-BRAF/EGFR therapy.

## Main

**Extended Data Fig. 1:**
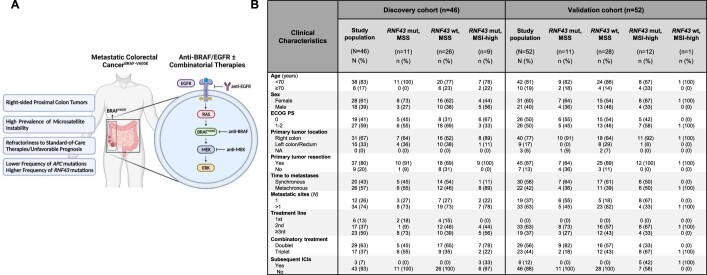
Patients’ characteristics. **A)** Patient and therapy flowchart. Specific clinical and genetic characteristics of mCRC^BRAF-V600E^ compared to mCRC^BRAF-wild-type^ tumors. Schematic description of the anti-BRAF/EGFR combinatorial therapies (doublet vs triplet) for patients with mCRC^BRAF-V600E^. **B)** Clinical characteristics of patients in the discovery (n = 46) and validation (n = 52) cohorts. Abbreviations: ECOG PS, Eastern Cooperative Oncology Group Performance Status; ICIs, immune checkpoint inhibitors; MSI-high, microsatellite instability high; MSS, microsatellite stable.

The﻿ criteria to match patients with cancer with the most effective therapies relies on the identification of molecular tumor dependencies that can be targeted with available treatments^1^. The *BRAF*^V600E^ mutation is found in approximately 10% of patients with metastatic colorectal cancer (mCRC), and its clinical presentation is often associated with a predominance of right-sided proximal tumors, high prevalence of microsatellite instability (MSI; near 30%), refractoriness to standard-of-care therapies and an unfavorable prognosis (Extended Data Fig. 1a)^2,3^_._ Compared to mCRC^BRAF-wild-type^, *BRAF*^V600E^ tumors (hereafter referred to as mCRC^BRAF-V600E^) also associate with specific molecular features, including a low frequency of *APC* mutations and a high rate of mutations in the tumor suppressor gene *RNF43* (refs. ^4,5^), a RING E3 ubiquitin ligase involved in suppression of the WNT–β-catenin pathway through promoting the degradation of FZD/WNT receptors^6,7^.

BRAF^V600E^ ATP-competitive kinase inhibitors were designed and clinically tested for the treatment of BRAF^V600E^-driven tumors, thus achieving variable outcomes depending on tumor type^8^. In particular, patients with melanoma harboring *BRAF*^V600E^ mutations have been demonstrated to derive marked benefit from BRAF inhibitor monotherapy (up to 70% objective response rate (ORR)^9,10^), while, in stark contrast, patients with *BRAF*^V600E^-mutant CRC receiving the same treatment experienced little clinical benefit^8,11,12^. Preclinical studies uncovered an intricate molecular circuitry in CRC^BRAF-V600E^ leading to rapid compensatory activation of the epidermal growth factor receptor (EGFR) that likely hampered the clinical outcomes of these patients^13,14^. These key findings set the rationale for the design of clinical trials targeting both BRAF and EGFR, with or without additional targeted therapies (that is, MEK, ERK or PIK3CA inhibitors) that generally achieved improved clinical outcomes as compared to previous standard-of-care treatments^12,15–17^.

The clinical outcomes of patients with mCRC^BRAF-V600E^ treated with the triplet regimen of encorafenib, cetuximab and binimetinib were assessed in the phase 3 BEACON CRC trial (NCT02928224). The study showed that combinatorial blockade of BRAF and EGFR, with or without concomitant MEK inhibition, achieved improved ORR (26% with the triplet and 20% with the doublet therapy versus 2% in the control arm) and extended overall survival (OS) and progression-free survival (PFS)^18,19^. These results warranted approval of the doublet therapy as a new standard therapy for CRC^BRAF-V600E^ by the US Food and Drug Administration and European Medicines Agency^20^. While the responses documented are unprecedented for patients with mCRC^BRAF-V600E^, these still compare unfavorably with the higher response rates observed in *BRAF*-mutant metastatic melanomas treated with anti-BRAF therapy^9,10^ and display a high degree of heterogeneity, which underscores the need for a deeper understanding of factors modulating treatment response that can optimize the clinical management of patients^1,21^.

**Fig. 1:**
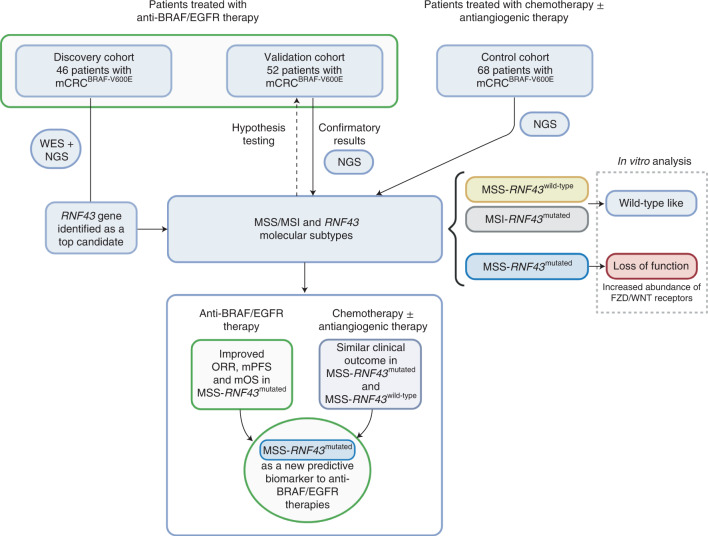
Study design. A total of 166 patients with mCRC^BRAF-V600E^ were included in the study from discovery (*n* = 46), validation (*n* = 52) and control (*n* = 68) cohorts. WES of germline DNA, baseline tumor DNA and/or baseline plasma cfDNA from 28 patients was performed. Targeted NGS was used to assess *RNF43* tumor mutation status for the 18 remaining patients from the discovery cohort and all tumors from the validation and control cohorts. Genomic profiles and MSS/MSI-*RNF43* molecular subtypes were compared with clinical response data (ORR, mPFS and mOS) using dNdScv maximum-likelihood unbiased mutation enrichment analysis^25^. In vitro assays were used to assess the functional impact of *RNF43* mutations detected in patient samples (see more in Fig. 6).

Herein we sought to explore genetic biomarkers with a predictive value that can contribute to refining the stratification of patients with mCRC^BRAF-V600E^ treated with anti-BRAF/EGFR combinatorial therapy. We applied whole-exome sequencing (WES) and/or targeted gene sequencing on baseline tumor and/or plasma cell-free DNA (cfDNA) samples from a large cohort of patients with mCRC^BRAF-V600E^ treated with anti-BRAF/EGFR therapy, as well as from a control cohort of patients with mCRC^BRAF-V600E^ receiving standard chemotherapies and antiangiogenic agents (and not exposed to anti-BRAF), and integrated these data with clinical correlates of response and survival. Our findings identified molecular subtypes based on microsatellite-stable (MSS)/MSI status and *RNF43* alterations and uncovered the predictive value of *RNF43* mutations as a biomarker of clinical outcome, including increased ORR, PFS and OS, to anti-BRAF/EGFR ± combinatorial therapies. Specifically, our data show that patients with MSS-mCRC^BRAF-V600E^ tumors harboring loss-of-function mutations in *RNF43* respond favorably to anti-BRAF/EGFR combinatorial therapy, whereas those with functional *RNF43* derived limited benefit from this regimen (Fig. 1).

## Results

### Description of patient cohorts treated with anti-BRAF/EGFR

A total of 98 patients with mCRC^BRAF-V600E^ treated with anti-BRAF/EGFR ± combinatorial therapies in clinical trials or who received such therapies through a compassionate use program were included in the current study (Fig. 1 and Extended Data Fig. 1b). The discovery cohort was composed of 46 patients from the Vall d’Hebron University Hospital (Barcelona, Spain) prospectively included from 2013 to 2021, and the validation cohort comprised 52 patients from three academic hospitals from Italy (Fondazione IRCCS Istituto Nazionale dei Tumori, Milan, Italy; University Hospital of Pisa, Pisa, Italy; Istituto Oncologico Veneto IOV-IRCCS, Padova, Italy).

**Fig. 2:**
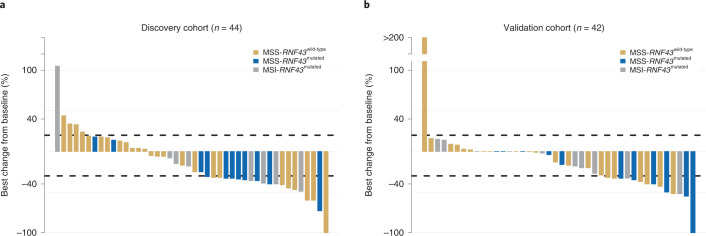
Clinical responses to BRAF/EGFR inhibition according to MSS/MSI and *RNF43* status. **a**,**b**, Waterfall plots representing best observed response in the mCRC^BRAF-V600E^ discovery cohort (*n* = 44) (**a**) and validation cohort (*n* = 42) (**b**), measured as percentage best change from baseline according to RECIST 1.1 criteria^22^ (Methods). Colors refer to molecular subtypes: MSS-*RNF43*^wild-type^ tumors (yellow), MSS-*RNF43*^mutated^ tumors (blue) and MSI-*RNF43*^mutated^ tumors (gray).

In the discovery cohort, 28 patients (61%) were female, with a median age at diagnosis of 61 years (range, 33–82 years), 31 patients (67%) had right-sided tumors, 34 patients (74%) had more than one metastatic site, and 37 patients (80%) had MSS and 9 patients (20%) had MSI tumors. In total, 35 patients (76%) received anti-BRAF/EGFR-based combinations as second- or third-line therapy, 6 patients (13%) received this as first-line therapy and 5 patients (11%) received this beyond third-line therapy. The number of patients who received the doublet combination was 29 (63%), and 17 patients (37%) received the triplet combination. The ORR by Response Evaluation Criteria in Solid Tumors version 1.1 (RECIST 1.1; ref. ^22^) was 44% (20/45 patients, one patient was not evaluable for response). The number of patients with MSI tumors who received immunotherapy after targeted therapy was 3 (7%). The *RNF43* mutation frequency was 43% (20/46) in the overall discovery cohort, 100% (9/9, all G659fs) in MSI tumors and 30% (11/37) in MSS tumors (Supplementary Table 1 and Fig. 2a).

In the validation cohort, 31 patients (60%) were female, with a median age at diagnosis of 62 years (range, 38–80 years), 40 patients (77%) had right-sided tumors, 33 patients (63%) had more than one metastatic site, and 39 patients (75%) had MSS and 13 patients (25%) had MSI tumors. Overall, 33 (63%) patients received anti-BRAF/EGFR-based combinations as second-line therapy, and 19 patients (37%) received this as third-line therapy. The number of patients who received the doublet combination was 29 (56%), and 23 patients (44%) received the triplet combination. The ORR by RECIST 1.1 (ref. ^22^) was 27% (14/51 patients, one patient was not evaluable for response). Six patients (12%) with MSI tumors received immunotherapy after targeted therapy. The *RNF43* mutation frequency was 44% (23/52) in the overall validation cohort, 92% (12/13, G659fs) in MSI tumors and 28% (11/39) in MSS tumors (Supplementary Table 1 and Fig. 2b).

### Biomarkers of response to anti-BRAF/EGFR in MSS-mCRC^BRAF-V600E^

**Extended Data Fig. 2:**
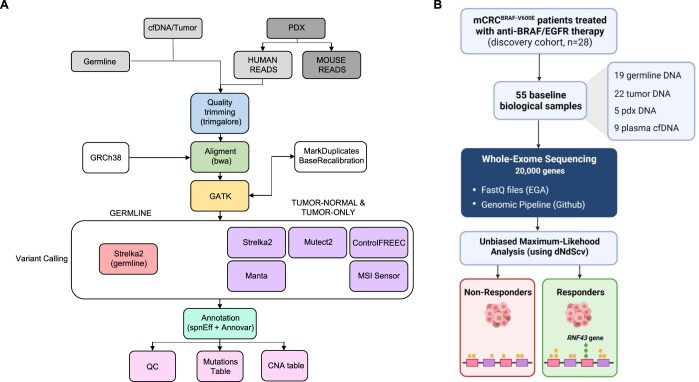
Bioinformatics pipeline for genomic data analysis. **A)** Description of the Sarek bioinformatics pipeline, a Nextflow-based pipeline that integrates all the processing, mapping, variant calling, and QC steps^40^, used for the WES analysis. **B)** WES based on responders vs non-responders from the discovery cohort (n = 28) followed by dNdScv^25^ maximum-likelihood unbiased mutation enrichment analysis was used as the genomic biomarker discovery strategy on 55 biological samples from mCRC^BRAF-V600E^ patients collected at baseline to anti-BRAF/EGFR ± MEKi therapies. The FastQ files have been deposited in the European Genome-phenome Archive (EGA, https://ega-archive.org) biorepository. The code of the bioinformatics pipelines can be found at https://github.com/nf-core/sarek and at https://github.com/jfnavarro/scitron. Abbreviations: cfDNA, cell-free DNA; CNA, copy number alteration; QC, quality control; PDX, patient-derived xenograft.

**Extended Data Fig. 3:**
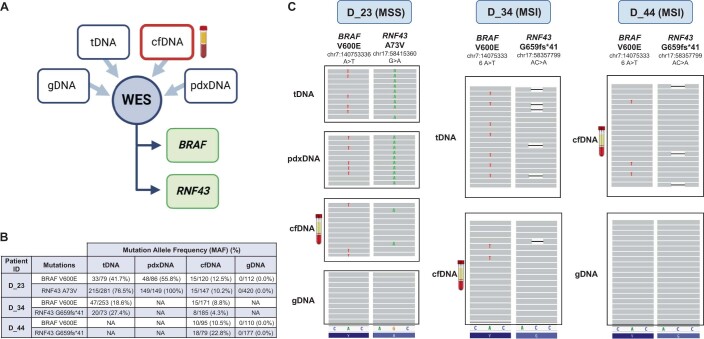
The utility of liquid biopsy to assess *BRAF* and *RNF43* mutational status in patiets with mCRC^BRAF-V600E^ treated with anti-BRAF/EGFR combinatorial therapies. **A)** WES was performed in 55 baseline biological samples from 19 gDNA, 22 tDNA, 9 cfDNA, and 5 pdxDNA from 28 patients with mCRC^BRAF-V600E^ from the discovery cohort treated with anti-BRAF/EGFR combinatorial therapies. The presence of the *BRAF*^V600E^ and *RNF43* mutations was interrogated in tumor-derived and plasma-derived samples. **B)** Mutation allele frequencies (MAF) of the *BRAF* and *RNF43* specific mutations are shown to gDNA, tDNA, and pdxDNA from patients with multiple samples which are available. **C)** *BRAF*^V600E^ and *RNF43* mutations were detected not only in tDNA and/or pdxDNA but also in available cfDNA samples, suggesting the utility of liquid biopsy for the assessment of these biomarkers. Abbreviations: gDNA, germline DNA; tDNA, tumor DNA; cfDNA, cell-free DNA; MSI, microsatellite instability high; MSS, microsatellite stable; pdxDNA, patient-derived xenograft DNA.

WES analysis detected mutations in several cancer-related genes previously reported to be implicated in mCRC^BRAF-V600E^ tumors, such as *APC*, *TP53*, *ARID1A*, *PIK3CA*, *FBXW7* and *RNF43*, among others (Supplementary Table 2). In general, the mutation frequencies of the discovery cohort were comparable to those observed in The Cancer Genome Atlas (TCGA) CRC tumors carrying the *BRAF*^V600E^ mutation from the PanCancer Atlas^23,24^ (*n* = 46; Supplementary Table 2). Unbiased maximum-likelihood analysis of WES mutational data from responders (partial response (PR) and complete response (CR)) versus nonresponders (stable disease (SD) and progressive disease (PD)) using dNdScv^25^ identified the *RNF43* gene as a top candidate gene associated with ORR (*P* values and *q* values <0.001; Extended Data Fig. 2a,b). Other candidate genes were identified, but we focused on *RNF43* because of its high mutation frequency and implications in CRC biology. *RNF43* mutations were detected in both tumor-derived and plasma cfDNA-derived DNA samples (Extended Data Fig. 3a–c).

**Extended Data Fig. 4:**
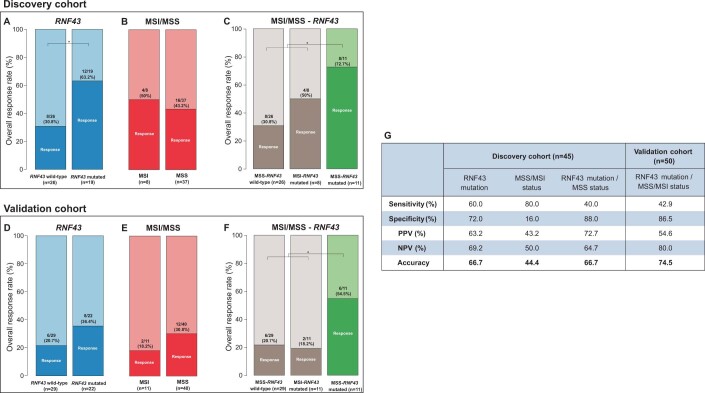
Overall response rate (ORR) (%) of the discovery (n = 45, A–C) and validation (n = 50, D–F) cohorts and diagnostic performance analyses (G) defined by MSS/MSI and *RNF43* mutation statuses. **A)** Patients with *RNF43*^mutated^ tumors exhibited an increased ORR (63%) compared to patients with *RNF43*^wild-type^ (31%). **B)** There were no significant differences between ORRs according to MSI/MSS status (50% and 43%, respectively). **C)** Interestingly, patients with mCRC^BRAF-V600E^ with MSS-*RNF43*^mutated^ tumors demonstrated increased ORR (73%) compared to the other groups, as MSI-*RNF43*^mutated^ and MSS-*RNF43*^wild-type^ (50% and 31%, respectively) (*P* = 0.03). Chi-square test was used for the statistical analysis. **D–F)** ORR of the validation cohort including 50 patients with mCRC^BRAF-V600E^ tumors treated with anti-BRAF/EGFR combinatorial therapies in 2nd or 3rd line. **D)** *RNF43*^mutated^ group exhibited an increased ORR (36%) compared to *RNF43*^wild-type^ group (21%). **E)** There were no significant differences between MSI/MSS status (31% and 17%, respectively). **F)** Interestingly, MSS-*RNF43*^mutated^ group showed increased ORR (54%) compared to the other groups, MSI-*RNF43*^mutated^ and MSS-*RNF43*^wild-type^ (18% and 21%), respectively (*P* = 0.02). Chi-square test was used for the statistical analysis﻿ (*P < 0.05). **G)** Table showing results of the accuracy, sensitivity, specificity, and positive (PPV) and negative predictive value (NPV) calculated to quantify the diagnostic performance of statuses of each potential biomarker (MSS/MSI and *RNF43* separated, and MSS/MSI-*RNF43* combined, in the discovery and validation cohorts). Abbreviations: MSI, microsatellite instability high; MSS, microsatellite stable.

The mutation rate of *RNF43* in the discovery cohort (20/46, 43%) was consistent with the rate reported in the TCGA PanCancer Atlas for CRC^BRAF-V600E^ (refs. ^23,24^; 21/46, 46%). To test the potential association between *RNF43* mutations and ORR, we analyzed data from the whole discovery series including WES and next-generation sequencing (NGS) panel data from 45 evaluable patients with mCRC^BRAF-V600E^. ORR in the whole *RNF43*^mutated^ subgroup, including MSS and MSI tumors, was 63% (12/19), which was significantly superior to the ORR of 31% achieved in patients with *RNF43*^wild-type^ status (8/26; *P* = 0.03; Extended Data Fig. 4a). The accuracy of *RNF43* mutations alone for predicting response to treatment was 67% (sensitivity, 60%; specificity, 72%; positive predictive value (PPV), 63%; negative predictive value (NPV), 69%; Extended Data Fig. 4g). In line with findings in the BEACON trial, patients with MSS and MSI CRC^BRAF-V600E^ achieved similar ORRs (50% (4/8) versus 43% (16/37); *P* = 1; Extended Data Fig. 4b), and the predictive accuracy of MSS/MSI status alone was 44% (sensitivity, 80%; specificity, 16%; PPV, 43%; NPV, 50%; Extended Data Fig. 4g).

We next classified tumor samples according to three molecular subtypes: MSS-*RNF43*^wild-type^, MSS-*RNF43*^mutated^ and MSI-*RNF43*^mutated^. The ORR of the MSS-*RNF43*^mutated^ subtype was significantly higher than those of the MSS-*RNF43*^wild-type^ and MSI-*RNF43*^mutated^ subtypes (73% versus 31% and 50%, respectively; *P* = 0.03) (Fig. 2a and Extended Data Fig. 4c). In agreement, accuracy for prediction of response combining MSS/MSI + *RNF43* mutation status was 67% (sensitivity, 40%; specificity, 88%; PPV, 73%; NPV, 65%; Extended Data Fig. 4g).

### Validation of MSS/MSI-*RNF43* status and clinical response

**Extended Data Fig. 5:**
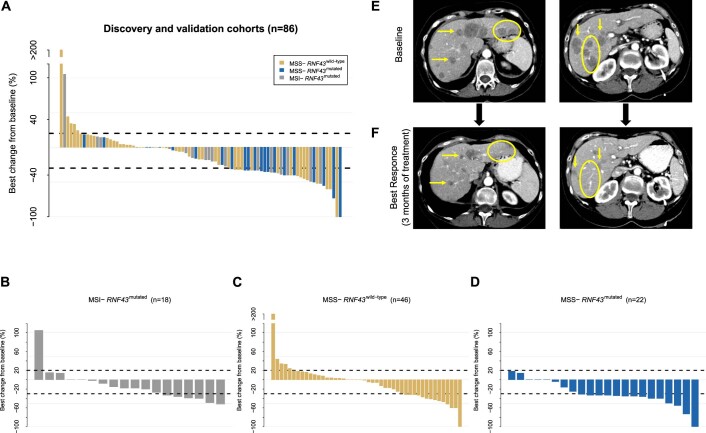
Waterfall plots depicting different molecular subtypes. **A)** Integrated waterfall plot showing best change in the total diameter of target lesions from baseline in 86 patients with mCRC^BRAF-V600E^ tumors treated with anti-BRAF/EGFR combinatory therapies (discovery cohort, n = 44, plus validation cohort, n = 42). As a common in real-world clinical practice for aggressive tumors like mCRC^BRAF-V600E^, images from a proportion of patients (n = 12) were not available, mostly due to rapid clinical deterioration. **B–D)** Waterfall plots including patients with the different molecular subtypes: **B)** MSI-*RNF43*^mutated^ tumors are shown in gray, **C)** MSS-*RNF43*^wild-type^ tumors are shown in yellow, and **D)** MSS-*RNF43*^mutated^ tumors are shown in blue. **E)** The post-contrast CT scan at baseline shows multiple liver metastases (yellow arrows) in a patient with mCRC^BRAF-V600E^ with a MSS-*RNF43*^mutated^ tumor. **F)** The post-contrast CT scan after 3 months of treatment shows marked reduction in size of the liver metastases (yellow arrows); some of the lesions are no longer seen in the follow-up CT-scan (yellow circles). Abbreviations: MSI, microsatellite instability high; MSS, microsatellite stable.

To explore the generalizability of the results obtained in the discovery set, we sought to validate the predictive value of *RNF43* mutations in an external independent cohort of patients treated with anti-BRAF/EGFR ± combinatorial therapies as second- or third-line therapy (*n* = 52). Overall, the ORR in the validation cohort was 27% (95% confidence interval (CI), 16.3–42%; 14/51 patients) with a median PFS (mPFS) of 4.4 months (95% CI, 4.1–5.9 months) and a median OS (mOS) of 8.5 months (95% CI, 6.9–14.2 months) (Supplementary Table 1). Consistent with the results in the discovery cohort, patients in the MSS-*RNF43*^mutated^ subtype in the validation cohort also achieved a significatively higher ORR compared to those in the MSS-*RNF43*^wild-type^ and MSI-*RNF43*^mutated^ subtypes (54% versus 21% and 18%, respectively; *P* = 0.02; Fig. 2b and Extended Data Figs. 4d–f and 5a–d).

**Fig. 3:**
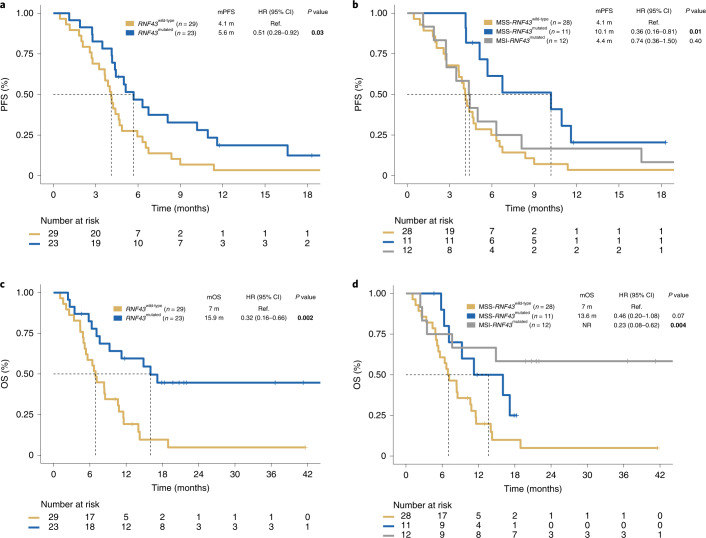
Survival analysis of patients undergoing BRAF/EGFR inhibition according to MSS/MSI and *RNF43* status. **a**,**b**, Kaplan–Meier curves representing PFS of patients with *RNF43*^wild-type^ (*n* = 29) and *RNF43*^mutated^ (*n* = 23) tumors (**a**) and combined *RNF43* and MSS/MSI status (MSS-*RNF43*^wild-type^ (*n* = 28), MSS-*RNF43*^mutated^ (*n* = 11) and MSI-*RNF43*^mutated^ (*n* = 12)) (**b**). **c**,**d**, OS of patients with *RNF43*^wild-type^ (*n* = 29) and *RNF43*^mutated^ (*n* = 23) tumors (**c**) and combined *RNF43* and MSS/MSI status (MSS-*RNF43*^wild-type^ (*n* = 28), MSS-*RNF43*^mutated^ (*n* = 11) and MSI-*RNF43*^mutated^ (*n* = 12)) (**d**). Cox models were used to obtain HRs with 95% CIs, and the two-sided log-rank test was used for statistical comparisons without adjustment for multiplicity. Colors indicate molecular subtypes: *RNF43*^wild-type^ tumors with or without MSS (yellow), *RNF43*^mutated^ tumors with or without MSS (blue) and MSI-*RNF43*^mutated^ tumors (gray). Significant values are shown in bold. m, months; NR, not reported; Ref., reference.

**Fig. 4:**
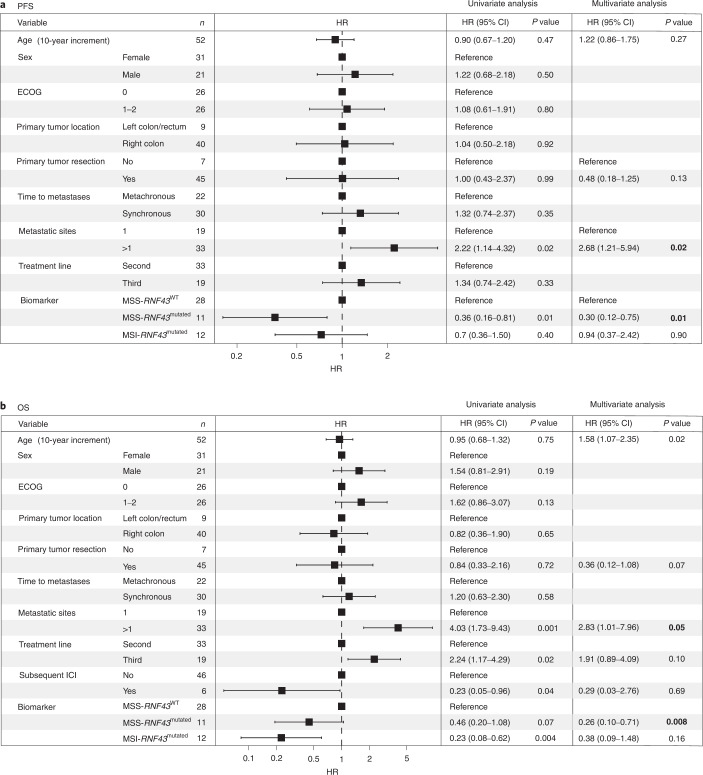
Univariate and multivariate Cox regression models. **a**,**b**, Analysis performed in the validation cohort (*n* = 52) for PFS (**a**) and OS (**b**). Univariate HRs along with 95% CIs are represented for each prognostic factor. *P* values were estimated by means of the two-sided log-rank test in the univariate analysis and by means of the Cox model in the multivariate analysis (two-sided). Significant values are shown in bold. ECOG, Eastern Cooperative Oncology Group performance; ICI, immune checkpoint inhibitor.

**Extended Data Fig. 6:**
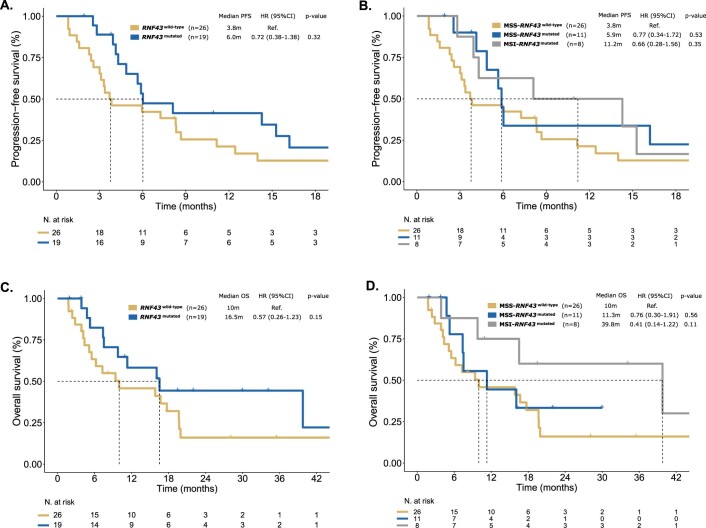
Kaplan-Meier curves of PFS and OS from patients with mCRC^BRAF-V600E^ tumors (discovery cohort, n = 45) treated with anti-BRAF/EGFR combinatory therapies with regard to *RNF43* mutations (left) and *RNF43* mutations combined with MSS/MSI status (right). **A)** PFS of patients with *RNF43*^mutated^ (n = 19) and *RNF43*^wildtype^ (n = 26) tumors and **B)** combined *RNF43* and MSS/MSI statuses (MSS-*RNF43*^wild-type^ [n = 26], MSS-*RNF43*^mutated^ [n = 11], MSI-*RNF43*^mutated^ [n = 8]). **C)** Event-free survival of patients with *RNF43*^mutated^ (n = 19) and *RNF43*^wild-type^ (n = 26) tumors and **D)** combined *RNF43* and MSS/MSI statuses (MSS-*RNF43*^wild-type^ [n = 26], MSS-*RNF43*^mutated^ [n = 11], MSI-*RNF43*^mutated^ [n = 8]). Cox models were used to obtain hazard ratios with 95% CIs, and the two-sided log-rank test was used for statistical comparisons without adjustment for multiplicity. Colors indicate molecular subtypes: RNF43^wild-type^ tumors with or without MSS (yellow), RNF43^mutated^ tumors with or without MSS (blue) and MSI-RNF43^mutated^ tumors (gray). Significant values are shown in bold. Abbreviations: CI, confidence interval; HR, hazard ratio.

We next evaluated whether the observed increased ORR was a surrogate for improved outcomes measured by PFS and OS. The mPFS in the MSS-*RNF43*^mutated^ subtype was 10.1 months (hazard ratio (HR), 0.36; 95% CI, 0.16–0.81), 4.1 months in the MSS-*RNF43*^wild-type^ subtype and 4.4 months (HR, 0.74; 95% CI, 0.36–1.50) in the MSI-*RNF43*^mutated^ subtype (Fig. 3a,b and Extended Data Fig. 6a,b). The MSS-*RNF43*^mutated^ subtype also showed a trend toward better OS compared to the MSS-*RNF43*^wild-type^ subtype (mOS of 13.6 versus 7 months; *P* = 0.07; HR, 0.46; 95% CI, 0.20–1.08; Fig. 3c,d and Extended Data Fig. 6c,d). In multivariate analysis, the MSS-*RNF43*^mutated^ subtype maintained an independent association with OS (HR, 0.26; 95% CI, 0.10–0.71; *P* = 0.008) after adjusting for imbalance in prognostic factors such as age and metastatic site (Fig. 4a,b). Despite having a short PFS in response to anti-BRAF/EGFR therapies, patients with MSI-*RNF43*^mutated^ status did not show a significantly lower mOS when compared to those with MSS-*RNF43*^mutated^ status, most likely because of the positive impact of the treatment with immune-checkpoint inhibitors administered in six patients (12%) with MSI after progression on anti-BRAF/EGFR therapy (HR, 0.38; 95% CI, 0.09–1.48; *P* = 0.16; Fig. 4b).

We also sought to explore whether, in addition to their power to predict response, the MSS/MSI-*RNF43* molecular subtypes could anticipate refractoriness to anti-BRAF/EGFR ± combinatorial therapies. Of the 16 patients with refractory mCRC^BRAF-V600E^ in the discovery and validation cohorts with PD as the best observed response, 13 (13/16, 81%) were MSS-*RNF43*^wild-type^, 3 (3/16, 19%) were MSI-*RNF43*^mutated^ and none (0/16, 0%) were MSS-*RNF43*^mutated^. Strikingly, none of the 22 patients with MSS-*RNF43*^mutated^ status in the two cohorts had PD as the best response (Extended Data Fig. 5d).

### Predictive value of *RNF43* mutations in MSS-mCRC^BRAF-V600E^

**Fig. 5:**
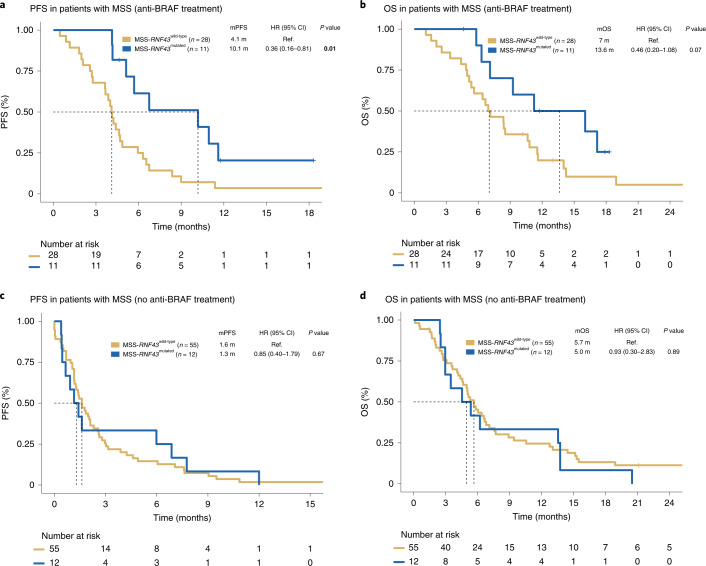
Predictive value of MSS/MSI-*RNF43* status. **a**–**d**, Kaplan–Meier curves representing PFS (**a**) and OS (**b**) for patients with mCRC^BRAF-V600E^ undergoing anti-BRAF/EGFR therapy in the validation cohort (anti-BRAF/EGFR as second or third line, *n* = 39) and PFS (**c**) and OS (**d**) in patients in the control cohort not exposed to BRAF inhibition (*n* = 67 second and third treatment lines; Extended Data Fig.7﻿). Cox models were used to obtain HRs with 95% CIs, and the two-sided log-rank test was used for statistical comparisons without adjustment for multiplicity. Colors refer to molecular subtypes: MSS-*RNF43*^wild-type^ (yellow) and MSS-*RNF43*^mutated^ (blue). Significant values are shown in bold.

**Extended Data Fig. 7:**
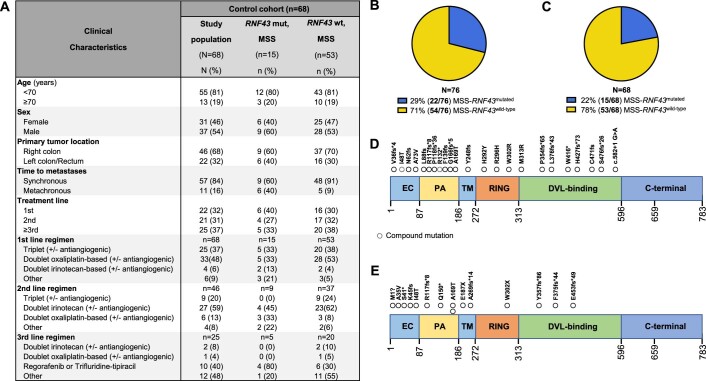
Clinical characteristics and mutational profile of patients with mCRC^BRAF-V600E^ treated with no anti-BRAF/EGFR combinatorial therapies. **A)** Clinical and therapeutic characteristics of patients from the control cohort, patients receiving standard chemotherapy ± antiangiogenic agents (total N = 68). These patients received in total 135 chemotherapy regimens with/without anti-angiogenic drugs during first, second, or third lines. A total of 67 treatment lines (second and third line) in MSS patients were analyzed. **B**–**E)** Comparison of *RNF43* mutation frequencies and protein location between discovery/validation (**B,D**) and control **(C,E)** cohorts. *RNF43* mutation frequencies of the mCRC^BRAF-V600E^ MSS subgroup from discovery/validation (29%, 22/76) and control (22%, 15/68) cohorts were similar (**B**, **C**), respectively. Also, the MSS discovery/validation and control cohorts showed a similar *RNF43* mutational profile regarding localization and predictive functional status (**D, E**). Abbreviations: MSS, microsatellite stable.

**Extended Data Fig. 8:**
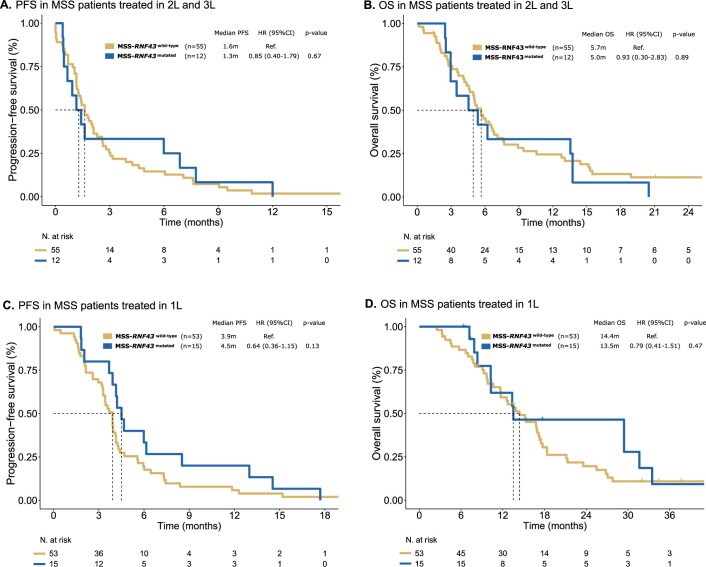
Analysis of the prognostic/predictive value of *RNF43* mutations in the control cohort of patients with mCRC^BRAF-V600E^ with MSS tumors treated with standard-of-care regimens only, with no exposure to anti-BRAF therapies. **A, B)** Kaplan-Meier curves of PFS and OS from the control cohort treated in second or third line (n = 67) **C, D)** and in first line (n = 68) with standard-of-care regimens only, with no exposure to anti-BRAF therapies. Colors refer to molecular subtypes:﻿ *RNF43*^wild-type^ (yellow), ﻿*RNF43*^mutated^ tumors (blue)^wild-type^. Cox models were used to obtain hazard ratios with 95% CI, and the two-sided log-rank test was used for statistical comparisons without adjustment for multiplicity. Abbreviations: CI, confidence interval; HR, hazard ratio.

To further confirm the value of *RNF43* mutations in predicting response to treatment in patients with MSS-mCRC^BRAF-V600E^ tumors, we aggregated data from 68 sex- and age-matched patients treated in four different hospitals (Extended Data Fig. 7a–e). All patients received standard-of-care chemotherapies and antiangiogenic agents that did not involve anti-BRAF therapy. The median age at diagnosis was 60.5 years (range, 30–80 years); 68% of patients had right-sided tumors. With regard to MSS/MSI-*RNF43* status, 22% of patients were MSS-*RNF43*^mutated^ and 78% were MSS-*RNF43*^wild-type^, similar to the discovery and validation MSS tumor cohorts (29% and 71%, respectively) (Extended Data ﻿Fig. 7a–c and Supplementary Table 1). Moreover, the MSS discovery/validation and control cohorts presented with similar *RNF43* mutation frequencies (29% (22/76) and 22% (15/68), respectively) (Extended Data Fig. 7b,c) as well as *RNF43* mutation localization patterns (Extended Data Fig. 7d,e). Among 68 patients treated in the second- and third-line setting, those in the MSS-*RNF43*^mutated^ subgroup achieved an mPFS of 1.3 months as compared to 1.6 months in the MSS-*RNF43*^wild-type^ population (HR, 0.85; 95% CI, 0.40–1.79; *P* = 0.67). Similarly, we found no differences in mOS after the start of second-line therapy (5 months in patients with MSS-*RNF43*^mutated^ tumors and 5.7 months in patients with MSS-*RNF43*^wild-type^ tumors; HR, 0.93; 95% CI, 0.30–2.83; *P* = 0.89) (Fig. 5a–d). To corroborate these findings, we also evaluated mPFS in the first-line setting for the entire control cohort and found no significant differences in the MSS-*RNF43*^mutated^ versus MSS-*RNF43*^wild-type^ population (4.5 months versus 3.9 months, respectively; HR, 0.64; 95% CI, 0.36–1.15; *P* = 0.13) (Extended Data Fig. 8a–d). Altogether, these data suggest that *RNF43* mutations have no prognostic relevance in patients with MSS-mCRC^BRAF-V600E^ in our cohort and support their predictive value in response to anti-BRAF/EGFR combinatorial therapies in this clinical setting. Conversely, the predictive value of *RNF43* mutations seen in MSS tumors was not observed in MSI tumors.

### Molecular profiles of *RNF43* mutations in mCRC^BRAF-V600E^

**Fig. 6:**
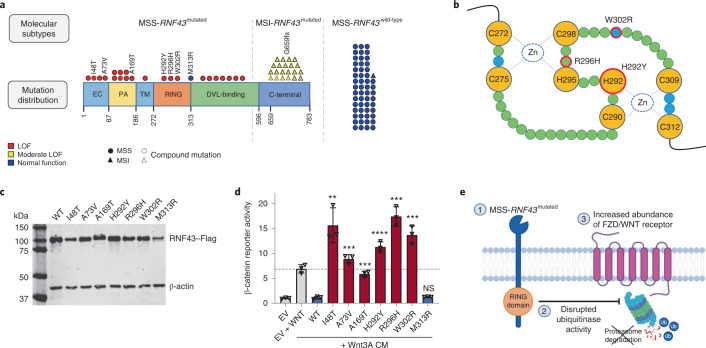
Functional analysis of *RNF43* mutations. **a**, Distinct localization and functional impact of *RNF43* mutations among patients with mCRC^BRAF-V600E^ treated with anti-BRAF/EGFR therapy according to MSS/MSI and *RNF43* molecular status (*n* = 98). MSI CRCs (*n* = 21) carried a mutation encoding G659fs, while MSS CRCs were divided into MSS-*RNF43*^mutated^ (*n* = 22) and MSS-*RNF43*^wild-type^ (*n* = 54) tumors. Numbers indicate the amino acid residue in the RNF43 protein sequence. The MSS-*RNF43*^mutated^ subtype harbored mutations mainly in the RNF43 N-terminal domain. Colors indicate the effect of the mutation on protein function: loss of function (LOF) (red), moderate loss of function (yellow) and normal function (blue); symbols reflect MSS/MSI status (circle and triangle, respectively) and the presence of a compound mutation (discontinuous border). **b**, Illustration of the ‘cross-brace’ topology of the RING domain containing a highly conserved sequence of cysteine–histidine residues that coordinates two atoms of zinc and four hydrophobic residues that are involved in binding to E2 (canonical sequence, CX_2_CX_(9–39)_CX_(1–3)_HX_(2–3)_CX_2_CX_(4–48)_CX_2_C). Figure adapted from ref. ^36^, Frontiers Media (CC BY 4.0 license). Mutations encoding H292Y, R296H and W302R in the RING domain of RNF43 are circled in red, while the mutation encoding M313R is located right outside the RING protein domain and likely for this reason did not affect RNF43’s ubiquitinase function. The four conserved residues are shown in blue. **c**, Western blot quantification of Flag-tagged RNF43 protein ectopically expressed in the HEK293T cell line; β-actin, loading control. This experiment was repeated twice obtaining the same protein expression levels. **d**, In vitro luciferase reporter assays representing levels of β-catenin activation (*y* axis) upon ectopic expression of RNF43 mutants (*x* axis) in HEK293T cells, following stimulation with Wnt3A conditioned medium (CM). One representative experiment is shown. The assay was performed three times with basically identical results. The statistical significance of all mutants relative to wild-type protein was obtained using a two-sided Student’s *t*-test (**P < 0.01, ***P < 0.001, ****P < 0.0001; NS, not significant). Empty vector (EV) and EV + WNT (control conditions) are shown in gray, *RNF43* alterations that behaved as loss-of-function variants are shown in red and wild-type protein and the M313R variant are shown in blue. **e**, Graphical representation of the impact of *RNF43* mutations (1) in impairing the ubiquitinase activity of the protein (2), resulting in the accumulation of FZD/WNT receptors in the cell membrane (3). Ub, ubiquitin. Source data

To better understand the underlying molecular features of MSS-*RNF43*^mutated^ CRC^BRAF-V600E^ tumors, we next classified and characterized the observed *RNF43* somatic mutations occurring in patients from both the discovery and validation cohorts. *RNF43* mutations were identified in 21 of 22 (95%) MSI tumors and 22 of 76 (29%) MSS tumors. In addition to the different frequencies, the location and functional nature of the *RNF43* mutations were fundamentally distinct in MSI and MSS tumors (Fig. 6a). All MSI *RNF43*-mutated tumors carried a hotspot mutation encoding p.G659fs*41 at the C-terminal, which is caused by a DNA slippage error at the seven-guanine repeat region, typical in MSI tumors lacking full DNA repair machinery, and classified as moderate loss of function with retained wild-type-like functional properties^26^. Conversely, the mutations occurring in MSS tumors were not recurrent and spanned the N-terminal domain of the protein, including the extracellular (EC), protease-associated (PA), transmembrane (TM), RING finger (RING) and DVL2-binding (DVL) domains. We identified 7 missense mutations in these domains and 16 that led to protein truncations predicted to cause loss of function^27,28^ (Fig. 6a,b). To validate the biological impact of the *RNF43* missense mutations detected in the MSS-mCRC^BRAF-V600E^ tumors of our patients, we performed luciferase reporter assays^27,28^. Compared to wild-type *RNF43*, six of seven (86%) of the *RNF43* mutations analyzed (encoding I48T, A73V, A169T, H292Y, R296H and W302R substitutions) behaved as loss-of-function variants (Fig. 6c,d). The corresponding inactivated RNF43 mutants lost the capacity to ubiquitinate and degrade FZD/WNT receptors, resulting in an accumulation of these receptors on the cell membrane and in high WNT signal levels in the luciferase reporter assays (Fig. 6c–e). Because of this effect, *RNF43* loss-of-function mutations are also referred to as ‘WNT-hyperactivating’ mutations^26–28^.

**Extended Data Fig. 9:**
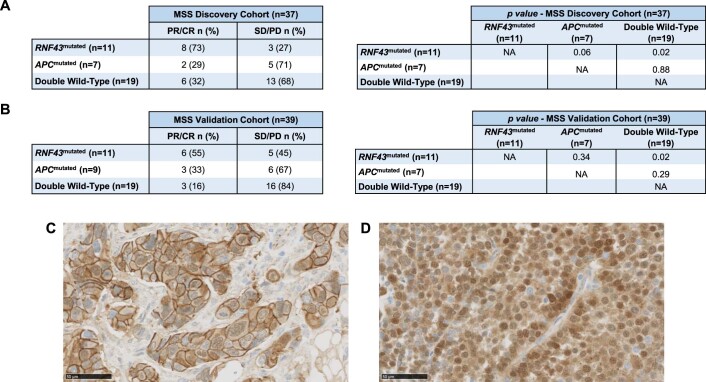
Mutation frequencies of *RNF43* and *APC* genes in MSS status and immunohistochemical analysis against human β-catenin of mCRC^BRAF-V600E^ tumor samples. **A, B)** Mutation frequencies of *RNF43* and *APC* genes, and response status from patients with mCRC^BRAF-V600E^ with MSS tumors from the discovery (**A**) and validation (**B**) cohorts. Chi-square test was used for the statistical analysis. Abbreviations: CR, complete response; MSS, microsatellite stable; PD, progressive disease; PR, partial response; SD, stable disease. **C–D)** Immunohistochemical analysis against human β-catenin was performed in MSS-*RNF43*^wild-type^, MSS-*RNF43*^mutated^, and MSI-*RNF43*^mutated^ mCRC^BRAF-V600E^ tumor samples (n = 33) to search for potential differential pattern in total expression or cellular localization of β-catenin protein. No correlation was found between β-catenin protein expression levels (0, no staining; 1, weak; 2, moderate; and 3, strong) and cellular localization (cytoplasm, membrane, nucleus) with response to anti-BRAF/EGFR ± combinatorial therapies. Representative immunohistochemistry for β-catenin with strong membrane positivity, weak-moderate cytoplasmic positivity, and few weak nucleus positivity (magnification 40x) (**C**) and strong-moderate nuclear and cytoplasmic positivity **(D)** (magnification 40x).

Mutations detected in other genes of the WNT pathway, such as *APC* and *CTNNB1*, are shown in Supplementary Table 1 and did not correlate with ORR (Extended Data Fig. 9a,b). Moreover, no correlation was observed of β-catenin protein expression levels and localization with response to anti-BRAF/EGFR ± combinatorial therapies, MSS/MSI status or *RNF43* mutation status in 33 mCRC^BRAF-V600E^ tumors from the discovery cohort (Extended Data Fig. 9c,d).

## Discussion

mCRC^BRAF-V600E^ represents an entity of its own with particular phenotypic features and with crucial implications in terms of prognosis^2,3^. Hence, there is a critical need for new clinical–biological insights that can lead to therapeutic improvements and the identification of new biomarkers of response. However, identification of biomarkers in this patient population, as well as development of new therapeutic strategies, has been challenging in this particular tumor type, given its underrepresentation in randomized clinical trials due to its low frequency (up to 12% of mCRC) and the high tumor burden and poor clinical conditions that impair inclusion of these patients in clinical trials^29^. Indeed, some of the current recommendations for standard-of-care treatments are based on subgroup analyses of phase 3 clinical trials^3,30,31^. On the other hand, despite the meaningful clinical activity observed in clinical trials evaluating BRAF inhibitor-based combinations, not all patients respond the same, and some responses are relatively short. This disparity in terms of treatment benefit highlights the biological heterogeneity of mCRC^BRAF-V600E^ and justifies better molecular characterization to optimize treatment outcomes.

Through pursuing an unbiased genomic analysis of responders versus nonresponders, we detected a strong signal in the *RNF43* locus in association with clinical outcome in response to anti-BRAF/EGFR-based therapies that was confirmed in a validation cohort (Fig. 3a–d) but not observed in a sex- and age-matched control cohort of patients receiving standard chemotherapy ± antiangiogenic agents (Fig. 5a–d and Extended Data Fig. 8a–d). Our study uncovers a previously unknown value of *RNF43* mutations, occurring in 29% of MSS-mCRC^BRAF-V600E^ tumors, in predicting response and clinical outcome. In contrast to patients with MSI mCRC, the patient population with MSS-mCRC^BRAF-V600E^ currently lacks biomarkers to guide treatment decision-making. Although previous analyses had associated tumor-based transcription subtypes with response^32^, our study reports a potential genomic prediction biomarker to anti-BRAF/EGFR-based combinations in the patient population with mCRC^BRAF-V600E^. Specifically, we found that within the MSS group, which represents 70% of patients with mCRC^BRAF-V600E^, the occurrence of a *RNF43* mutation was associated with improved ORR and, importantly, longer PFS and OS with anti-BRAF/EGFR strategies (Figs. 2a,b, 3a–d﻿ and 5a–d and Extended Data Figs. 4a–g and 5a–f).

Overall, this finding has two main implications. First, incorporating *RNF43* mutation as a routine biomarker could contribute to defining the optimal treatment sequence in patients with MSS-mCRC^BRAF-V600E^ according to their predicted response profile. Second, it uncovers a cross-talk between MAPK and RNF43–WNT pathways in the antitumor activity of BRAF/EGFR-targeting therapy, which might be exploited as a future potential therapeutic target.

The mechanistic basis underlying the clinical success of concomitant BRAF/EGFR inhibition builds upon preclinical evidence of a rapid feedback compensation for EGFR mediated by the activation of CRAF or by the transactivation of CRAF–BRAF heterodimers^13,14^. Our findings underscoring the potential role of RNF43 in modulating response to anti-BRAF/EGFR therapy point to an interplay between the MAPK and WNT signaling pathways in MSS-mCRC^BRAF-V600E^ tumors. Specifically, our in vitro experiments show that most *RNF43* mutations detected in MSS tumors of responding patients have a loss-of-function effect, which contrasts with *RNF43* mutations described in MSI tumors, where the protein retains its function^26^. In our patient datasets, loss of RNF43 would impair degradation of WNT/FZD receptors, leading to activation of the WNT pathway. While the molecular intricacies through which the MAPK and WNT pathways cross-talk to modulate the antitumor activity of this treatment combination need to be studied in detail, our clinical findings are consistent with preclinical studies describing a role for WNT activation in antagonizing MAPK-driven proliferation to preserve an equilibrium with differentiation of intestinal stem cells^33^. It is therefore plausible to speculate that a similar mechanism of RNF43 loss-dependent WNT activation may be restraining MAPK signaling in MSS mCRC tumors and synergizing with pharmacological blockade of the pathway. Of note, we found no correlation of response with the presence of *APC* or *CTNNB1* mutations or β-catenin protein expression or localization in mCRC^BRAF-V600E^ tumors (Extended Data Fig. 9a–d and Supplementary Table 1), suggesting that noncanonical WNT pathways^34,35^, rather than canonical (β-catenin-dependent) signaling, might be involved in modulation of anti-BRAF/EGFR activity.

Despite our study representing, to our knowledge, the largest genetic biomarker analysis to date on this patient population, the overall number of patients remains limited owing to the rarity of *BRAF*^V600E^ mutation in mCRC and the difficulties in accessing clinically annotated cohorts with comprehensive genomic profiling. Furthermore, while our genomic analyses uncover a robust association of *RNF43* mutations with clinical outcome, the inherent heterogeneity of our real-world patient cohorts receiving treatment in four different hospitals and undergoing molecular profiling with different multigene panels represents another limitation to be addressed in prospective, standardized biomarker studies. The lack of randomized data represents a constraint for analysis of the predictive versus prognostic role of *RNF43* mutations. In this regard, our efforts to identify control patients exposed to chemotherapies and antiangiogenic agents during the same time period and at the same hospitals that contributed data for the discovery and validation cohorts are the best mitigation available, also taking into account that similar NGS platforms were used for molecular stratification. Finally, despite our in vitro analysis supporting a loss-of-function effect of most *RNF43* mutations detected in MSS tumors from responding patients, the mechanistic basis for how altered WNT signaling modulates MAPK pathway activation in response to anti-BRAF/EGFR agents remains to be fully elucidated.

In summary, *RNF43* mutations represent a new biomarker that warrants further validation for its potential to help prioritize anti-EGFR/BRAF combinations in selected patients with mCRC^BRAF-V600E^ who are more likely to derive benefit and identify those patients for whom alternative treatment approaches are needed. Future research should explore incorporating this biomarker in routine testing along with *BRAF* and MSS/MSI status and evaluate their integration with other transcriptomic, microbiome or microenvironmental indicators for optimizing the clinical management of this heterogeneous and complex disease.

## Methods

### Predictive and prognostic value analysis

To assess the predictive value of *RNF43* status, we aggregated the clinical and genetic data of 68 patients from four different cohorts (hospitals participating in the present work) treated with standard-of-care regimens only (no exposure to anti-BRAF therapies) whose tumors harbored *BRAF*^V600E^ mutation and with information on the status of MSI and *RNF43* mutations. These patients received in total 135 chemotherapy regimens with or without antiangiogenic drugs during the first, second or third line of therapy. To control for potential confounding factors, we excluded (1) treatments in the first line and (2) patients whose tumors were MSI and who received anti-PD1/PD-L1 therapies during the disease course. A total of 67 treatment lines (second and third line) in patients with MSS were analyzed. To compare the prognosis of patients with MSS-*RNF43*^mutated^ and MSS-*RNF43*^wild-type^ tumors, PFS and OS endpoints were estimated using the Kaplan–Meier method. Mixed-effects Cox models considering patient ID as a random effect, to adjust for the intra-patient variability in patients with more than one line of therapy, were used to obtain HRs with 95% CIs (Supplementary Table 1).

### Ethics committee approval

The study was approved by each investigational site’s institutional review board/ethics committee: Vall d’Hebron Institute of Oncology (VHIO), Barcelona, Spain; Veneto Institute of Oncology IRCCS, Padova, Italy; Azienda Ospedaliero-Universitaria Pisana, University of Pisa, Pisa, Italy; and Fondazione IRCCS Istituto Nazionale dei Tumori, Milan, Italy. The research was conducted in accordance with the Declaration of Helsinki and local data protection laws. All patients were provided with written informed consent before enrollment. All data provided are anonymized in line with applicable laws and regulations.

### Radiological response evaluation

All cases were reviewed by board-certified radiologists with experience in oncology imaging and clinical trials. Radiological response for each patient was classified following the principles of RECIST 1.1 (ref. ^22^): CR (disappearance of the lesion or reduction in the short axis to <10 mm in the case of a pathological lymph node), PR (a decrease by at least 30% in the long axis for visceral or soft tissue disease and the short axis for pathological lymph nodes), PD (an increase of at least 20% in the long axis in the case of visceral or soft tissue disease and the short axis in the case of pathological lymph nodes) or SD (when the lesion did not fulfill the criteria for PR or PD). As is commonly the case in clinical practice for aggressive tumors, some patients included in the study had no image available due to evident progression as per rapid clinical deterioration, increase in the levels of tumor markers in plasma (CEA) and progression of nontarget disease. These patients were considered to have clinical PD.

### DNA extraction

DNA extraction from tumor samples was performed with the automated system Maxwell 16 FFPE plus LEV DNA purification kit (Promega), and quality and concentration were measured with a NanoDrop 1000 spectrophotometer (Thermo Fisher Scientific). cfDNA was extracted from 1 ml of plasma using the QIAamp Circulating Nucleic Acid Kit (Qiagen), based on the manufacturer’s recommendations, and quantified using the highly sensitive kit for the Qubit, dsDNA HS (High Sensitivity) Assay Kit (Thermo Fisher Scientific).

### WES

WES was performed in 55 baseline biological samples available at VHIO’s sample biobank (19 germline DNA, 22 tumor DNA, 5 patient-derived xenograft DNA and 9 plasma cfDNA) from 28 patients from the discovery cohort. Genomic libraries were prepared using 10–15 ng of cfDNA and the ThruPLEX DNA or Plasma-seq Kit (Rubicon Genomics; now commercialized as the SMARTer ThruPLEX plasma-seq kit by Takara Bio)^37,38^. Quality control of libraries was carried out in TapeStation (Agilent), and amplified profiles of ~300 base pairs were considered for downstream analysis. Capture of the genomic coding regions for WES was performed using the SureSelect Human All Exon V5 or V6 kit (Agilent), with a target sequencing output of 12 Gb (100×) for genomic DNA and 36 Gb (300×) for tumor and cfDNA. Sequencing was carried out on a HiSeq or NovaSeq Illumina sequencing platform.

### NGS cancer gene panel

Tumor samples from the discovery cohort that did not have WES data (*n* = 18) and from the validation (*n* = 52) and control (*n* = 68) cohorts were genetically analyzed using a VHIO in-house NGS test of 430 cancer genes or a Foundation Medicine or Caris Life Sciences commercial NGS platform^39^.

### Bioinformatics

#### Data processing and analysis (WES)

The WES samples (FASTQ files) were processed using sarek (v2.7.1)^40^. Briefly, the following steps were performed: quality filtering and adaptor trimming with TrimGalore; alignment to the reference genome with BWA; marking of duplicates with GATK4 MarkDuplicates; recalibration of scores with GATK4 BaseRecalibrator and ApplyBSQR; and computing of pileups with SAMtools. Variant calling was performed using four different tools: Mutect2, Strelka2, MSIsensor and Control-FREEC (tumor-only and pair mode). Filtering of the variants generated by Mutect2 was conducted using GATK4 GetPileupSummaries, CalculateContamination and FilterMutectCalls. Annotation of the variants was performed using snpEff. MultipleQC and statistics were generated using FastQC, Qualimap, SAMtools, VCFtools and MultiQC. We used a public Panel of Normals (PON) obtained from the public repository of GATK at gs://gatk-best-practices/somatic-hg38/1000g_pon.hg38.vcf.gz and a BED file containing all the genomic intervals of the kits used to generate the WES samples. Mutect2 variants corresponding to the tumor-only samples were refiltered with GATK4 FilterMutectCalls using a value of 10 for the parameter ‘-max-events-in-region’. These variants were subsequently reannotated with snpEff with the same genome reference, version and settings that were used in the processing pipeline. The generated mutational data were subsequently summarized, postprocessed, filtered and validated for downstream analysis.

#### Mutational enrichment analysis

Two different analyses were performed on the discovery cohort to determine which genes were enriched for somatic mutations in the responder and nonresponder groups. In the first analysis, we summarized the number of mutations, accumulated mutated allele frequency and number of samples/patients per gene. In the second analysis, we computed somatic enrichment *P* values and *q* values for each group using dNdScv^25^ (GRCh38). This tool models the background mutation rate of each gene by combining local information (synonymous mutations in the gene) and global information (nonsynonymous mutations in the gene and other covariates) and controlling for the sequence composition of the gene and mutational signatures^25^.

### Functional classification of *RNF43* mutations

#### Functional classification derived from previous in vitro and in vivo studies

To initially assess the potential biological consequences of the identified *RNF43* mutations, we applied a recently developed functional classification derived from in vitro and in vivo studies^26–28^. Accordingly, *RNF43* mutations can be classified as (1) wild-type like, (2) activators of the WNT–β-catenin pathway or (3) hyperactivators of the WNT–β-catenin pathway^26–28^. Mechanistically, wild-type-like mutants maintain their capability to repress the WNT–β-catenin pathway and activators lose their ubiquitinase function, leading to an increased abundance of FZD/WNT receptors in the cellular membrane and activation of the WNT–β-catenin pathway, while hyperactivators exert a dominant-negative effect on the wild-type protein, completely blocking ubiquitination of FZD/WNT receptors and thereby leading to extremely high levels of WNT–β-catenin pathway activation^26–28^.

#### Luciferase reporter assays with *RNF43* mutation expression vectors

To further demonstrate the biological impact of the identified *RNF43* missense mutations, we performed luciferase reporter assays with ectopic expression of the *RNF43* mutants. Flag-tagged *RNF43* mutation expression plasmids were generated using the New England Biolabs Q5 Mutagenesis Kit^27,28^. All plasmids were full-length sequence verified.

HEK293T cells were used for the assays, cultured in DMEM (Gibco) supplemented with 10% (vol/vol) FBS (Gibco). Cells were cultured in a humidified incubator maintained at 37 °C with 5% CO_2_. Cells tested negative for mycoplasma based on the real-time PCR method at Eurofins GATC-Biotech (Konstanz, Germany). Identity of the HEK293T cells was confirmed by the Erasmus Molecular Diagnostics Department, using PowerPlex-16 STR genotyping (Promega). For the β-catenin reporter assays, HEK293T cells were seeded in 24-well plates and transfected with 100 ng WRE Wnt/β-catenin reporter, 100 ng RNF43 expression plasmid (wild-type RNF43, mutant RNF43 or empty vector) and 10 ng CMV-Renilla expression plasmid, using Lipofectamine 2000 (Thermo Fisher Scientific) as transfection reagent. L-Wnt3A or L-control conditioned medium (diluted 30-fold in normal growth medium) was added after 16 h. Cells were lysed in passive lysis buffer (Promega) 48 h after transfection. Next, the firefly and *Renilla* luciferase activity was measured with the Dual-Luciferase Reporter Assay System (Promega) using a LumiStar Optima luminescence counter (BMG LabTech). β-catenin reporter activity was measured in triplicate. All β-catenin reporter values were normalized to the value obtained for cells with empty vector exposed to L-control conditioned medium, which was arbitrarily set to 1. One representative experiment is shown. The assay was performed three times with basically identical results. Proper expression of Flag-tagged RNF43 was analyzed using fluorescent western blotting as described below.

#### Immunoblotting analysis

Immunoblotting was carried out using standard methods. HEK293T cells transfected with RNF43 were lysed in 2× Laemmli sample buffer with 0.1 M dithiothreitol and heated for 10 min at 95 °C. Proteins were separated by 10% SDS–PAGE and then transferred to an Immobilon-P PVDF membrane (MilliporeSigma). The membrane was blocked for 1 h with Odyssey Blocking Buffer (LI-COR Biosciences) at room temperature and incubated overnight with primary antibodies at 4 °C. After washing three times with 0.05% Tween-20 in TBS (TBST) buffer for 10 min, the membrane was incubated for 1 h with IRDye 680 Goat Anti-Mouse (1:10,000; LI 926-68070, LI-COR Biosciences) and then washed three times with TBST for 10 min. The membrane was scanned on the Odyssey Infrared Imaging System (LI-COR Biosciences). Primary antibodies used were mouse anti-FLAG (1:1,000; F1804, Sigma-Aldrich) and mouse anti-β-actin (1:1,000; sc-47778, Santa Cruz).

#### Immunostaining of β-catenin in CRC^BRAF-V600E^ tumors

Immunohistochemical analysis was performed using a primary antibody against human β-catenin (prediluted, Beta-Catenin Mouse Monoclonal Antibody, clone 14, 760-4242, Cell Marque) in Benchmark ULTRA (Ventana Medical Systems). Labeling was performed using ULTRA Cell Conditioning 1 (ULTRACC1) (950-224, Roche) antigen retrieval buffer for 64 min at 95 °C, followed by incubation with primary antibody for 32 min at 36 °C and detection with the Ventana UltraView Universal DAB Detection Kit (760-500, Roche). Immunohistochemistry was scored semiquantitatively by one pathologist (R.F.) using an H-score (for the cytoplasm, membrane and nucleus separately). H-score was obtained by multiplying the proportion of cells showing cytoplasmic, membrane or nuclear staining and the intensity of staining (0, no staining; 1, weak; 2, moderate; 3, strong).

#### Statistical analysis

A descriptive analysis of all included variables in the study was performed. Continuous variables were expressed as the median and interquartile range, and categorical variables were expressed as absolute values and percentages. ORR was estimated in all subgroups based on RECIST version 1.1 criteria along with 95% CIs, and Fisher’s exact test was used to assess statistical significance. Accuracy, sensitivity, specificity, and PPV and NPV were calculated to quantify the diagnostic performance of each potential biomarker.

PFS was defined as the time from anti-BRAF/EGFR therapy initiation to disease progression or death, whichever occurred first. OS was defined as the time from anti-BRAF/EGFR therapy initiation to death from any cause. PFS and OS were estimated using the Kaplan–Meier method and compared by the log-rank test. Cox proportional-hazard models were used to obtain HRs with 95% CIs. Formal statistical testing was only used in the validation cohort. No data imputation was performed. All tests were two sided with a value of *P* < 0.05 considered statistically significant without adjustment for multiple comparisons. All statistical analyses were performed using R statistical software.

### Reporting summary

Further information on research design is available in the Nature Research Reporting Summary linked to this article.

## Online content

Any methods, additional references, Nature Research reporting summaries, source data, extended data, supplementary information, acknowledgements, peer review information; details of author contributions and competing interests; and statements of data and code availability are available at 10.1038/s41591-022-01976-z.

## Supplementary information

Reporting Summary

Supplementary Tables Supplementary Table 1: Clinical and genetic dataset of mCRC^BRAF-V600E^ from the discovery and validation cohorts. Supplementary Table 2: Comparison of mutation frequencies of cancer-related genes from the discovery cohort and patients with mCRC^BRAF-V600E^ from the TCGA PanCancer Atlas.

## Data Availability

Reference genome GRCh38 was used for alignment. The FASTQ files corresponding to the WES data from clinical samples analyzed in the paper have been deposited in the European Genome-phenome Archive (EGA, https://ega-archive.org) under study ID EGAS00001006247 and dataset ID EGAD00001008755 with appropriate measures for controlled access (DUO: 0000020, DUO: 0000021). Further information about EGA can be found on https://ega-archive.org, “The European Genome-phenome Archive in 2021” (https://academic.oup.com/nar/advance-article/doi/10.1093/nar/gkab1059/6430505). Clinical and genetic data for the patients are available in Supplementary Table 1. Source data are provided with this paper.

## Source data

Source Data Fig. 6 Original unprocessed western blot from Fig. 6c.

## Code availability

The code for the pipeline that was used to process all samples can be found at https://github.com/nf-core/sarek. Sarek is a Nextflow-based pipeline that integrates all the processing, mapping, variant calling and quality control steps^25^. The code used for postprocessing, filtering, validation and analysis of the mutational data is available at https://github.com/jfnavarro/scitron.
