# Supplementary material for: RNF43 mutations predict response to anti-BRAF/EGFR combinatory therapies in BRAFV600E metastatic colorectal cancer
Source: Nat Med. 2022 Sep 12;28(10):2162–70. doi: 10.1038/s41591-022-01976-z (PMC9556333; doi:10.1038/s41591-022-01976-z)
Supplement: Supplementary file 1 — Reporting Summary [file 41591_2022_1976_MOESM1_ESM.pdf]

nature portfolio

Corresponding author(s): Rodrigo A. Toledo, Elena Elez

Last updated by author(s): Jul 13, 2022

Reporting Summary

Nature Portfolio wishes to improve the reproducibility of the work that we publish. This form provides structure for consistency and transparency in reporting. For further information on Nature Portfolio policies, see our Editorial Policies and the Editorial Policy Checklist.

Statistics

For all statistical analyses, confirm that the following items are present in the figure legend, table legend, main text, or Methods section.

n/a	Confirmed	
☐	☒	The exact sample size (*n*) for each experimental group/condition, given as a discrete number and unit of measurement
☐	☒	A statement on whether measurements were taken from distinct samples or whether the same sample was measured repeatedly
☐	☒	The statistical test(s) used AND whether they are one- or two-sided<br>*Only common tests should be described solely by name; describe more complex techniques in the Methods section.*
☐	☒	A description of all covariates tested
☐	☒	A description of any assumptions or corrections, such as tests of normality and adjustment for multiple comparisons
☐	☒	A full description of the statistical parameters including central tendency (e.g. means) or other basic estimates (e.g. regression coefficient) AND variation (e.g. standard deviation) or associated estimates of uncertainty (e.g. confidence intervals)
☐	☒	For null hypothesis testing, the test statistic (e.g. *F*, *t*, *r*) with confidence intervals, effect sizes, degrees of freedom and *P* value noted<br>*Give P values as exact values whenever suitable.*
☒	☐	For Bayesian analysis, information on the choice of priors and Markov chain Monte Carlo settings
☒	☐	For hierarchical and complex designs, identification of the appropriate level for tests and full reporting of outcomes
☒	☐	Estimates of effect sizes (e.g. Cohen's *d*, Pearson's *r*), indicating how they were calculated

Our web collection on statistics for biologists contains articles on many of the points above.

Software and code

Policy information about availability of computer code

Data collection	No software was used for data collection.
Data analysis	The code of the pipeline that was used to process all the samples can be found at https://github.com/nf-core/sarek. Sarek is a Nextflow based pipeline that integrates all the processing, mapping, variant calling, and QC steps. The code used for post-processing, filtering, validation, and analysis of the mutational data is available at https://github.com/jfnavarro/scitron. All statistical analyses were performed using R statistical software version 4.1.2.

For manuscripts utilizing custom algorithms or software that are central to the research but not yet described in published literature, software must be made available to editors and reviewers. We strongly encourage code deposition in a community repository (e.g. GitHub). See the Nature Portfolio guidelines for submitting code & software for further information.

Data

Policy information about availability of data

All manuscripts must include a data availability statement. This statement should provide the following information, where applicable:

- Accession codes, unique identifiers, or web links for publicly available datasets
- A description of any restrictions on data availability
- For clinical datasets or third party data, please ensure that the statement adheres to our policy

Reference genome GRCh38 was used for the alignment. The FastQ files corresponding to the whole exome-sequencing (WES) data from clinical samples analyzed in the paper have been deposited in the European Genome-phenome Archive (EGA, https://ega-archive.org) biorepository under the ID dataset of Study ID: EGAS00001006247 and the Dataset ID: EGAD00001008755 with appropriate measures for controlled access (collaboration required (DUO: 0000020), ethics

approval required (DUO: 0000021). Further information about EGA can be found on https://ega-archive.org, "The European Genome-phenome Archive in 2021" (https://academic.oup.com/nar/advance-article/doi/10.1093/nar/gkab1059/6430505).

Field-specific reporting

Please select the one below that is the best fit for your research. If you are not sure, read the appropriate sections before making your selection.

☒ Life sciences ☐ Behavioural & social sciences ☐ Ecological, evolutionary & environmental sciences

For a reference copy of the document with all sections, see nature.com/documents/nr-reporting-summary-flat.pdf

Life sciences study design

All studies must disclose on these points even when the disclosure is negative.

Sample size

Not pre-specific sample size calculation was performed for this project. The study included all available mCRC BRAF-V600E patients with information in RNF43 gene. A total of 166 metastatic mCRC BRAF-V600E patients (98 treated with anti-BRAF/EGFR therapies and 68 treated with standard-of-care not including BRAFi therapies) were included in the current study. Of the treated with anti-BRAF/EGFR therapies, the discovery cohort was composed of 46 patients from the Vall d'Hebron University Hospital (Barcelona, Spain) prospectively included from 2013 to 2021, and the validation cohort comprised 52 patients from three academic hospitals from Italy (Fondazione IRCCS Istituto Nazionale dei Tumori, Milan, Italy; Azienda Ospedaliero-Universitaria Pisana, University Hospital of Pisa, Pisa, Italy; Istituto Oncologico Veneto IOV-IRCCS, Padova, Italy). Control cohort was composed by patients from all the above referred institutes. The validation cohort (n=52) had > 80% power to validate statistically and clinically significant differences in PFS and OS with hazard ratio of 0.45 or lower using two-sided 0.05 alpha error.

Data exclusions

No genomics data were excluded from the analyses.
One patient from the discovery cohort and another from the validation cohort were not evaluable for response and were excluded from the overall response rate (ORR) analysis.

Replication

The results obtained in the discovery cohort were confirmed in an external validation cohort.

Randomization

This was a retrospective study and no randomization was performed. The current study aims to explore the predictive value of RNF43 status (baseline characteristic) but not the efficacy of a specific treatment strategy. In this particular scenario, randomization is not strictly needed to generate the set of evidence.

Blinding

Blindness is not relevant to studies searching for potential biomarkers of response to anti-cancer therapies. Importantly, an unbiased positive selection analysis was applied using mutation data obtained from whole-exome sequencing (WES) of the tumors from the responders and not responders.
Specifically, the unbiased maximum-likelihood analysis of WES mutational data from responders vs non-responders using dNdScv identified the RNF43 gene as a top candidate gene associated with ORR (p- and q-values <0.001).

Reporting for specific materials, systems and methods

We require information from authors about some types of materials, experimental systems and methods used in many studies. Here, indicate whether each material, system or method listed is relevant to your study. If you are not sure if a list item applies to your research, read the appropriate section before selecting a response.

Materials & experimental systems

n/a	Involved in the study	
☐	☒	Antibodies
☐	☒	Eukaryotic cell lines
☒	☐	Palaeontology and archaeology
☒	☐	Animals and other organisms
☐	☒	Human research participants
☐	☒	Clinical data
☒	☐	Dual use research of concern

Methods

n/a	Involved in the study	
☒	☐	ChIP-seq
☒	☐	Flow cytometry
☒	☐	MRI-based neuroimaging

Antibodies

Antibodies used

Anti-FLAG Antibody (1:1000, cat.# F1804, Sigma-Aldrich, St. Louis, MO, USA); Mouse anti-β-actin (1:1000, cat.# sc-47778, Santa Cruz, CA, USA); IRDye 680 goat anti-mouse (1:10.000, cat.# LI 926-68070, LI-COR Biosciences, Lincoln, NE, USA); human β-catenin Antibody (prediluted, cat.# 760-4242, lot V0002678, Beta-Catenin Mouse Monoclonal Antibody, clone 14, Cell Marque, Rocklin, CA, USA); Ventana UltraView Universal DAB Detection Kit (cat.#. 760-500, Roche, Penzberg, Germany).

Validation

Anti-FLAG antibody (https://scholar.google.com/scholar?hl=en&as_sdt=0%2C5&as_vis=1&q=f1804+Flag+sigma&btnG=); Mouse anti-β-actin (https://www.scbt.com/es/p/beta-actin-antibody-c4); IRDye 680 goat anti-mouse (https://scholar.google.com/scholar?hl=en&as_sdt=0%2C5&as_vis=1&q=IRDye+680+goat+anti-mouse+LI+926-68070&btnG=); betacatenin mouse monoclonal antibody

(https://www.cellmarque.com/antibodies/CM/21/Beta-Catenin_14); Ventana UltraView Universal DAB Detection Kit (https://scholar.google.es/scholar?hl=es&as_sdt=0%2C5&q=Ventana+UltraView+Universal+DAB+Detection+Kit+ref.+760-500+&btnG=).

Eukaryotic cell lines

Policy information about cell lines

Cell line source(s)	HEK293T has been cultured in the Erasmus MC-University Medical Center, Rotterdam, the Netherlands for decades and no specific origin is reported.
Authentication	Identity of HEK293T cell line was confirmed by the Erasmus Molecular Diagnostics Department, using Powerplex-16 STR genotyping (Promega, Leiden, The Netherlands).
Mycoplasma contamination	The HEK293T cell line tested negative for mycoplasma based on the real-time PCR method at Eurofins GATC-Biotech (Konstanz, Germany).
Commonly misidentified lines (See ICLAC register)	None

Human research participants

Policy information about studies involving human research participants

Population characteristics	A total of 166 patients with metastatic CRC-BRAF-V600E patients were included in the current study. 98 patients were treated with anti-BRAF/EGFR therapies: 46 patients belonged to the discovery cohort: female, 28/46 (61%) and male, 18/46 (39%) and the median age at diagnosis was 61 years old (range: 33-82) and 52 patients belonged to the validation cohort: female, 31/52 (60%) and male 21/52 (40%) and the median age at diagnosis was 62 years old (range: 38-80). Moreover, 68 patients were treated with non-BRAFi standard-of-care therapies (control cohort): female, 31/68 (46%) and male, 37/68 (54%) and median age at diagnosis 60.5 years old (range: 30-80).
Recruitment	Clinical data and samples from BRAF-V600E-mutated mCRC patients from the Vall d'Hebron Institute of Oncology (VHIO), Barcelona, Spain; Veneto Institute of Oncology IRCCS, Padova, Italy; Azienda Ospedaliero-Universitaria Pisana, University of Pisa, Pisa, Italy and Fondazione IRCCS Istituto Nazionale dei Tumori, Milan, Italy were retrospectively obtained and included in the study. There was no relevant bias on the inclusion of these patients that could affect the further analyses.
Ethics oversight	The study was approved by each investigational site's institutional review board/ethics com-mittee: Vall d'Hebron Institute of Oncology (VHIO), Barcelona, Spain; Veneto Institute of Oncology IRCCS, Padova, Italy; Azienda Ospedaliero-Universitaria Pisana, University of Pisa, Pisa, Italy and Fondazione IRCCS Istituto Nazionale dei Tumori, Milan, Italy. The research was conducted in accordance with the Declaration of Helsinki and local data protection laws. All patients provided written informed consent be-fore enrollment. All data provided are anonymized in line with applicable laws and regulations.

Note that full information on the approval of the study protocol must also be provided in the manuscript.

Clinical data

Policy information about clinical studies
All manuscripts should comply with the ICMJE guidelines for publication of clinical research and a completed CONSORT checklist must be included with all submissions.

Clinical trial registration	This was a translational research study, not a formal clinical trial.
Study protocol	This was a translational research study, not a formal clinical trial.
Data collection	Patients, genetics and clinical information from each center were retrospectively collected and data integrated for the study.
Outcomes	The evaluable outcomes were overall response rates (ORR), progression-free survival (PFS), and overall survival (OS).
