# Supplementary figures and images for: RNF43 mutations predict response to anti-BRAF/EGFR combinatory therapies in BRAFV600E metastatic colorectal cancer
Source: Nat Med. 2022 Sep 12;28(10):2162–70. doi: 10.1038/s41591-022-01976-z (PMC9556333; doi:10.1038/s41591-022-01976-z)

## Original IB image for Figure 6C

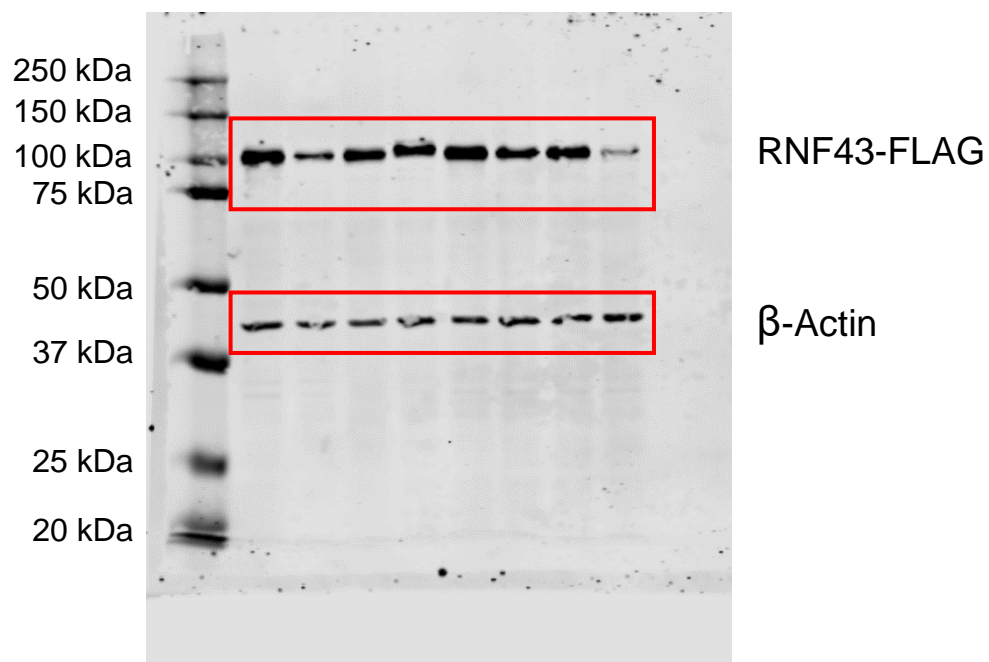

Supplement: Source Data Fig. 6 — Original unprocessed western blot from Fig. 6c. [file 41591_2022_1976_MOESM3_ESM.pdf]
